# Twelve-Month Results of a Single or Multiple Dexamethasone Intravitreal Implant for Macular Edema following Uncomplicated Phacoemulsification

**DOI:** 10.1155/2015/362564

**Published:** 2015-10-05

**Authors:** Solmaz Abdolrahimzadeh, Vito Fenicia, Maurizio Maurizi Enrici, Pasquale Plateroti, Dora Cianfrone, Santi Maria Recupero

**Affiliations:** ^1^Ophthalmology Unit, DAI Testa/Collo, Azienda Policlinico Umberto I, University of Rome “Sapienza”, Viale del Policlinico 155, 00161 Rome, Italy; ^2^Ophthalmology Unit, Sant'Andrea Hospital, NESMOS Department, University of Rome “Sapienza”, Via di Grottarossa 1035-1039, 00189 Rome, Italy

## Abstract

The clinical efficacy of one or two intravitreal injections of a continued deliverance dexamethasone 700 *μ*g implant in ten patients with persistent macular edema following uncomplicated phacoemulsification was evaluated. Complete ophthalmological examination and spectral domain optical coherence tomography were carried out. Follow-up was at day 7 and months 1, 2, 4, 6, 8, and 12. At baseline mean best corrected visual acuity was 62 Early Treatment Diabetic Retinopathy Study Chart letters, which showed statistically significant improvement at each follow-up, except at month 6, to reach 79 letters at month 12 (*P* = 0.018). Prior to treatment mean central foveal thickness was 622 *μ*m, which showed statistically significant improvement at each follow-up to reach a mean value of 282 *μ*m (*P* = 0.012) at month 12. Five patients received a second dexamethasone implant at month 7. Two patients were excluded from the study at months 4 and 8. Intraocular pressure remained stable during the study period with the exception of mild increase in two patients requiring topical therapy. In conclusion there was statistically significant improvement of best corrected visual acuity and mean central foveal thickness with one or two intravitreal dexamethasone implants over 12 months.

## 1. Introduction

Cystoid macular edema (CME) or Irvine-Gass syndrome is the main motive for inauspicious visual acuity achievement following uncomplicated cataract extraction. The incidence of CME after phacoemulsification is reported between 0.1 and 2% [1, 2]. Numerous factors have been held accountable in the pathogenesis of CME but the phenomenon is still poorly understood. However, it has been suggested that macular edema arises due to increased vascular permeability following surgical procedures such as cataract removal and pars plana vitrectomy, which cause the release of prostaglandins and disruption of the blood-retinal barrier [3, 4]. Corticosteroids, nonsteroidal anti-inflammatory agents, and carbonic anhydrase inhibitors have been employed as common treatment procedures [3–5]. Recently, intravitreal administration of antivascular endothelial growth factor agents have also been tested [6]. Treatment is recommended only in patients with clinically significant macular edema, which is considered when visual acuity is 20/40 or less [7]. To date, there is no standard treatment protocol for the management of chronic pseudophakic CME.

Intravitreal pharmacological treatment has the advantage of bypassing the blood-ocular barriers. Furthermore, due to the particular anatomy of the eye, high intravitreal levels of drug can be obtained and the efficacy of treatment can be intensified by drug distribution close to the target site. The dexamethasone implant (Ozurdex, Allergan Inc., Irvine, CA, USA) is an innovative treatment alternative for noninfectious posterior uveitis and macular edema in retinal vein occlusion [8]. Diabetic macular edema and, recently, prostaglandin-induced CME have also been treated with agreeable results [9–11]. This biodegradable implant measures 6.5 mm × 0.45 mm and is composed of a matrix consisting of a copolymer of lactic and glycolic acids and dexamethasone which dissolves completely into H_2_O and CO_2_ leaving no remnants. It is injected through the pars plana with a monouse 22-gauge injector and postimplantation sutures are not necessary. It furnishes continued deliverance of dexamethasone where peak doses are supplied for 2 months ensued by a slower release, altogether lasting for 6 months and providing 700 *μ*g of dexamethasone [12].

The use of this implant has been reported in pseudophakic CME with short-term follow-up [13–16]. To our knowledge there are no reports of long-term results and the number of implants necessary in the management of pseudophakic CME. This study was carried out to evaluate the long-term clinical efficacy of the dexamethasone intravitreal implant in patients with persistent CME following uncomplicated phacoemulsification.

## 2. Materials and Methods

In the present study we assessed 10 patients who were diagnosed with CME due to decreased visual acuity and increase in central foveal thickness (CFT) ensuing unremarkable phacoemulsification. The patients were unresponsive to topical steroids and nonsteroidal anti-inflammatory agents and received treatment with the dexamethasone implant at the Ophthalmology Unit of the St. Andrea Hospital, University of Rome “Sapienza”. According to the declaration of Helsinki, at the time of recruitment, informed consent to take part in the study was read and signed by all patients.

Exclusion criteria comprised patients with diabetes, uveitis, or other systemic diseases that could cause ocular involvement, patients who had undergone precedent surgical or parasurgical ocular procedures other than phacoemulsification, vitreomacular traction with epiretinal membrane or macular hole, age-related macular degeneration, retinal vascular pathologies, glaucoma, or elevated IOP. Furthermore, patients were also excluded if they were cortisone responders. Cataract extraction was performed with the divide and conquer technique and in-bag IOL implantation with no complications. Clinically significant CME was classified as visual acuity lower than 20/40 and CFT of more than 250 *μ*m persisting for a period longer than 90 days.

The method was similar to precedent studies on CME (14–16) and the following were carried out for all patients: ophthalmological examination comprising best corrected visual acuity (BCVA) assessment using Early Treatment Diabetic Retinopathy Study (ETDRS) charts, ICare Tonometry [17], and OCT evaluation using spectral domain optical coherence tomography (SD-OCT) evaluation (Cross Line, MM5, 3D Macular, RTVue SD-OCT) with CFT measurement.

The intravitreal dexamethasone implant was injected in the operating theatre through a biplanar intrascleral path with a 22-gauge needle. All patients were then examined at day 7 and months 1, 2, 4, 6, 8, and 12. Patients requiring a second injection of dexamethasone were implanted at month 7.

### 2.1. Statistical Analysis

Statistical analysis was carried out with SPSS software package V.21 (SPSS, Inc., Chicago, Illinois, USA). Since the normality of data could not be assumed because of the small sample size, the (nonparametric) Wilcoxon signed-rank test was used to evaluate the differences in the median values of BCVA, CFT, and IOP between baseline and day 7 and months 1, 2, 4, 6, 8, and 12. A *P* value < 0.05 was considered significant, meaning that the median of the difference (i.e., baseline day 7 or baseline month 6) is not 0.

## 3. Results

Ten patients with persistent pseudophakic CME who received one or two implants of dexamethasone were selected. The details regarding patient characteristics are given in Table 1. The average persistence of macular edema before dexamethasone implantation was 3.1 months. One patient was excluded from the study due to arterial occlusion at month 4 and a second patient decided to discontinue follow-up at month 8.

**Figure 1:**
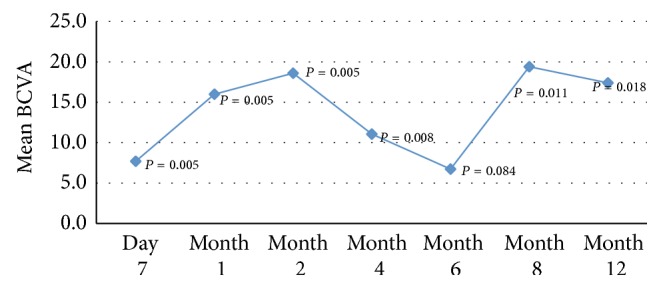
Mean change in best corrected visual acuity (BVCA) from baseline at each follow-up assessment.

Mean BCVA prior to treatment was 62 ETDRS letters. Following implantation, statistically significant improvement in BCVA was detected at day 7 and at each follow-up interval with the exception of month 6. Three patients did not require a second implant. In 5 eyes where visual acuity had declined and foveal thickness had increased, a second implant was injected at month 7. Mean BCVA was 79 ETDRS letters at 12 months (*P* = 0.018) (Table 1, Figure 1).

**Figure 2:**
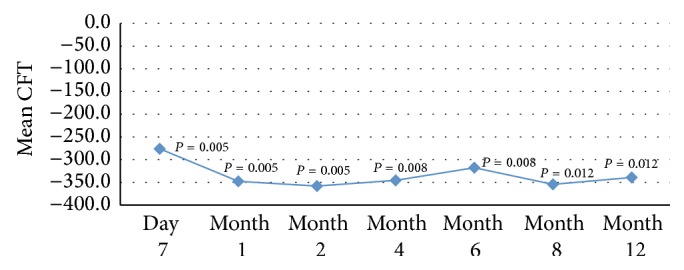
Mean change in central foveal thickness (CFT) from baseline at each follow-up assessment.

Prior to treatment the mean CFT was 622 *μ*m; following dexamethasone implantation, statistically significant improvement was seen at day 7 and at each follow-up interval to improve to 282 *μ*m (*P* = 0.012) at 12 months. A second implant was injected at month 7 in 5 patients who showed recurrence (Table 2, Figure 2).

**Figure 3:**
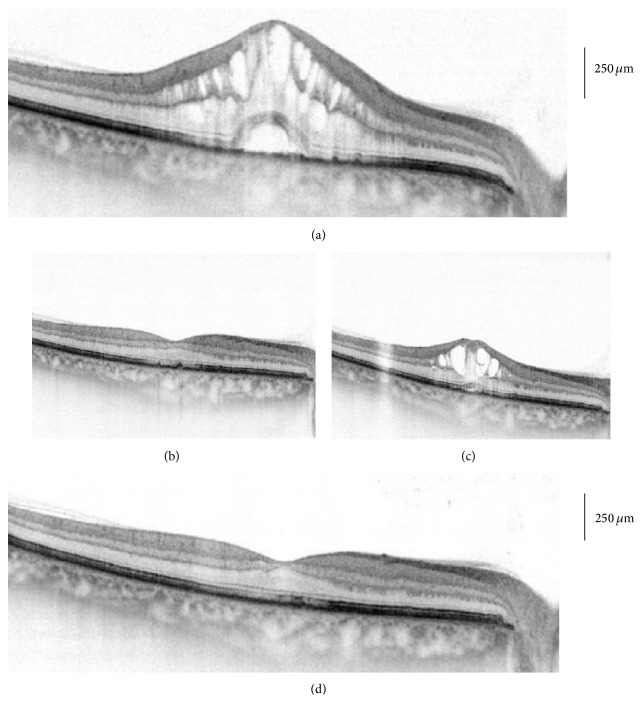
Optical coherence tomography of macular profile and thickness following dexamethasone implant at baseline and 7 months. (a) Before treatment, (b) 2 months following first dexamethasone implant, (c) 6 months following first dexamethasone implant, and (d) 12 months following second dexamethasone implant at month 7.

Figure 3 shows optical coherence tomography images of CFT change over time and results of a second implant in one patient with recurrence.

There were two cases of intraocular pressure increase >25 mmHg, which were successfully managed with topical timolol 0.5% and intraocular pressure remained stable during the study period (Table 3).

## 4. Discussion

In the present study on CME following uncomplicated phacoemulsification, mean BCVA and CFT improved following one or two injections of intravitreal dexamethasone implants over 12 months of follow-up. A second implant was required in five eyes whereas in three eyes results were maintained after only one implant throughout the follow-up period.

There have been few studies where the results of one intravitreal injection of dexamethasone have been evaluated with short-term follow-up in pseudophakic CME (13–16). Analysis of 8 patients with Irvine-Gass syndrome showed improvement of both BCVA and macular edema at 90 days from intravitreal injection of dexamethasone, which was maintained up to 180 days in 54% of eyes [13]. This is similar to our results where 50% of patients required a second implant after 6 months. Furino et al. and Al Zamil analyzed eyes with CME following uncomplicated phacoemulsification with a mean duration of 2.4 and 7.7 months, respectively, where a single injection of dexamethasone was performed and found significant reduction of macular edema and improvement of BCVA at 6 months [15, 16].

Medeiros et al. presented the outcome of a single dexamethasone implant in 9 patients with Irvine-Gass syndrome with a mean duration of 9.1 months. They reported peak effectiveness of the implant at 3 months from treatment and significant amelioration of macular thickness and BCVA during 6 months of follow-up [14]. In our study mean peak improvement of visual acuity was at 2 months following the first implant and at 8 months following the second implant. Mean peak improvement of CFT was at 2 months. Mean BCVA significantly improved at each follow-up except at month 6. According to the pharmacodynamics of the dexamethasone implant, this corresponds to the time when the release of dexamethasone is largely terminated. Nevertheless, at 12 months, following a second implant in 50% of patients, BCVA was significantly improved. As regarding CFT, there was a statistically significant improvement at each follow-up and the trend was more stable with respect to BCVA; however, even here there was a slight increase in CFT at 6 months (304 *μ*m).

Corticosteroids diminish the amount of intraocular prostaglandins and other factors believed to have a role in postoperative CME. In a prospective randomized controlled trial Negi et al. compared topical and periocular corticosteroids following routine cataract surgery and found both safe and effective routes of administration [18]. However, topical and periocular administration have a short half-life and have been reported to reach significant systemic concentrations [19]. Intravitreal injection of triamcinolone acetonide has been demonstrated to produce visual improvement and reduction of CME [20, 21] but the use of triamcinolone is limited due to the requirement of multiple injections and the side effects in terms of increase in intraocular pressure and cataract formation [22], although even with the dexamethasone implant a repeat injection was required in 50% of our cases. While dexamethasone is a more potent corticosteroid, it has a shorter half-life, which restricts its clinical efficacy as an injectable suspension, thus the rationale for an implant with a continued deliverance method, which can provide drug release through long periods with a minor frequency of adverse effects.

In two patients there was an increase in IOP > 25 which was successfully managed with topical treatment. This is in agreement with the reported trend following intravitreal dexamethasone administration where IOP is increased in 15% of patients with peak values at 60 days and returns to baseline at 6 months [23]. Patient 3 had retinal artery occlusion at month 4. We cannot be sure of the cause; however, in the judgment of the authors, it is unlikely that there was a reasonable possibility that this serious adverse effect was caused by the dexamethasone implant, the applicator, or the injection procedure. Furthermore, to our knowledge, arterial occlusion has not been reported in large patient population studies, which have also addressed the safety profile of the dexamethasone implant [9, 24]. There were no cases of cataract formation in any patient.

The limitations of our study were the retrospective description, the modest number of eyes, and the absence of a control group. The strength of our study was the long follow-up, which demonstrated the necessity for a second implant after 6 months in 50% of cases.

## 5. Conclusions

This is the first report on the long-term clinical results of the dexamethasone intravitreal implant in patients presenting CME following phacoemulsification. BCVA and CFT significantly improved at 12 months of follow-up although a second implant was required after 6 months in 50% of cases. Therefore, our studies suggest that single or multiple injections of the dexamethasone implant are an effective treatment option for patients with persistent CME ensuing uncomplicated cataract extraction with phacoemulsification.

## Figures and Tables

**Table 1:** Patient characteristics and best corrected visual acuity (BCVA) values in study eye using ETDRS charts prior to treatment and at follow-up intervals.

Patient	Gender	Age	Baseline	Day 7	Mo. 1	Mo. 2	Mo. 4	Mo. 6	Mo. 8	Mo. 12
1	F	72	78	84	86	87	88	88	88	88
2	F	81	71	73	78	79	80	80	80	80
3^§^	M	78	36	58	70	78	—	—	—	—
4^§^	M	77	35	36	73	78	49	36	—	—
5	F	78	70	71	73	74	76	78	83	84
6^*^	M	81	65	80	83	84	75	66	78	65
7^*^	F	62	72	74	80	83	76	70	80	78
8^*^	F	65	59	60	70	73	68	67	72	74
9^*^	M	71	60	78	80	83	67	62	83	75
10^*^	M	71	70	79	83	83	75	68	84	88

Mean		73.6	61.6	69.3	77.6	80.2	72.7	68.3	81	79
Median		74.5	67.5	73.5	79	81	75	68	81.5	79
SD		6.5	14.9	14.4	5.8	4.5	10.8	14.6	4.8	7.8
Range		62–81	35–78	36–84	70–86	73–87	49–88	36–88	72–88	65–8
*P* value^†^				**0.005**	**0.005**	**0.005**	**0.008**	0.084	**0.011**	**0.018**
Delta^‡^				7.7	16	18.6	11.1	6.7	19.4	17.4

^*^Patients denoted with ∗ had a second implant at month 7.

^§^Patients dropped out from the study.

^†^ *P* value refers to Wilcoxon signed-rank test on the median values with respect to the baseline.

^‡^Delta denotes the mean difference with the baseline.

Mo.: month.

**Table 2:** Central foveal thickness (CFT) prior to treatment and at follow-up intervals.

Patient	Baseline	Day 7	Mo. 1	Mo. 2	Mo. 4	Mo. 6	Mo. 8	Mo. 12
1	563	349	349	346	345	339	340	346
2	438	381	266	256	250	255	251	248
3^§^	852	361	221	220	—	—	—	—
4^§^	707	395	223	215	260	470	—	—
5	658	263	254	201	213	213	210	215
6^*^	630	312	279	266	295	309	250	369
7^*^	424	297	269	289	268	277	250	261
8^*^	808	397	270	263	278	280	273	268
9^*^	610	369	302	289	285	298	281	303
10^*^	526	331	305	289	290	293	282	249

Mean	621.6	345.5	273.8	263.4	276	303.8	267.1	282.4
Median	620	355	269.5	264.5	278	293	262	264.5
SD	142.2	44.3	38.5	43.4	36.0	71.5	37.5	52.7
Range	424–852	263–397	221–349	201–346	213–345	213–470	210–340	215–369
*P* value^†^		**0.005**	**0.005**	**0.005**	**0.008**	**0.008**	**0.012**	**0.012**
Delta^‡^		−276	−348	−358	−346	−318	−354	−339

^*^Patients denoted with ∗ had a second implant at month 7.

^§^Patients dropped out from the study.

^†^ *P* value refers to Wilcoxon signed-rank test on the median values with respect to the baseline.

^‡^Delta denotes the mean difference with the baseline.

Mo.: month.

**Table 3:** Intraocular pressure (mmHg) prior to treatment and at follow-up intervals.

	Baseline	Day 7	Mo. 1	Mo. 2	Mo. 4	Mo 6	Mo. 8	Mo. 12
1	17	14	14	14	14	14	13	14
2	11	16	13	13	13	13	13	13
3^§^	14	17	16	13	—	—	—	—
4^§^	13	21	21	20	18^t^	14^t^	—	—
5	14	16	12	12	14	13	13	16
6^*^	14	16	14	16	16	18	18	16
7^*^	14	16	20	17^t^	18^t^	15^t^	16^t^	16^t^
8^*^	15	19	19	18	16	15	20	16
9^*^	15	17	16	17	17	16	16	14
10^*^	15	16	17	16	16	17	17	17

Mean	14.2	16.8	16.2	15.6	15.8	15	15.8	16
Median	14	16	16	16	16	15	16	16
SD	1.5	1.9	3.0	2.5	1.8	1.7	2.6	1.4
Range	11–17	14–21	12–21	12–20	13–18	13–18	13–20	13–17
*P* value^†^		**0.031**	0.120	0.165	0.091	0.228	0.158	0.222
Delta^‡^		2.6	2.0	1.4	1.6	0.8	1.6	1.8

^*^Patients denoted with ∗ had a second implant at month 7.

^§^Patients dropped out from the study.

^†^ *P* value refers to Wilcoxon signed-rank test on the median values with respect to the baseline.

^‡^Delta denotes the mean difference with the baseline.

^t^Patients who were prescribed topical intraocular pressure lowering medication.

Mo.: month.
